# Relationship Between Perfluoroalkyl Acids in Human Serum and Sjogren’s Syndrome: A Case–Control Study of Populations in Hangzhou, China

**DOI:** 10.3390/toxics12100764

**Published:** 2024-10-21

**Authors:** Yun Zhao, Shetuan Hu, Hangbiao Jin, Chuanbing Fan, Kaizhen Liao, Songzhao Zhang, Jing Xue

**Affiliations:** 1 Department of Rheumatology, Zhejiang University School of Medicine Second Affiliated Hospital, Hangzhou 310009, China; 2315115@zju.edu.cn; 2 Key Laboratory of Microbial Technology for Industrial Pollution Control of Zhejiang Province, College of Environment, Zhejiang University of Technology, Hangzhou 310032, China; shetuanhu001@163.com (S.H.); hangbiao102@163.com (H.J.); chuanbingfan001@163.com (C.F.); kaizhenliao001@163.com (K.L.); 3 Department of Laboratory Medicine, Zhejiang University School of Medicine Second Affiliated Hospital, Hangzhou 310009, China; 2196005@zju.edu.cn

**Keywords:** environmental pollutants, perfluoroalkyl acids, Sjogren’s syndrome, autoimmune disease

## Abstract

Exposure to perfluoroalkyl acids (PFAAs) has been found to elicit a range of detrimental effects on human health. However, limited research has investigated the impact of PFAAs on immunity and immune disorders such as Sjogren’s syndrome, with existing studies yielding inconsistent results. This study was conducted in Hangzhou, China, with an initial cohort comprising 156 healthy individuals and 162 patients diagnosed with Sjogren’s syndrome. We quantified serum levels of PFAAs and examined associations between PFAAs and both susceptibilities to the development of Sjogren’s syndrome and immune marker concentrations. Nine PFAAs were frequently detected in the serum, with perfluorooctanoate (PFOA) exhibiting the highest concentration, followed by perfluorooctanesulfonate (PFOS). Exposure to PFOA and perfluorotridecanoate (PFTrDA) was inversely associated with the disease. Furthermore, a negative correlation between PFOA and C-reactive protein (CRP) was observed. These findings suggest that exposure to specific PFAAs may impact the immune system and potentially influence the development of Sjogren’s syndrome.

## 1. Introduction

In recent decades, perfluoroalkyl acids (PFAAs) have been extensively utilized in a wide range of industrial and consumer products, including but not limited to carpets, textiles, and food packaging [1,2,3], owing to their exceptional thermal and chemical stability as well as remarkable hydrophilic and lipophilic properties [4]. The release of PFAAs into the environment occurs throughout their life cycle, starting from the initial factory production, continuing through transportation to customers, and ultimately ending at the final disposal site [5]. Dramatically, it is these excessive properties that have led to their widespread and persistent presence in almost all environmental media [6,7,8]. Furthermore, PFAAs can enter the human body through the consumption of contaminated food and water, inhalation of indoor air, and exposure to other contaminated sources [9]. Hence, PFAAs have frequently been detected in human serum samples [10]. Due to health concerns regarding exposure to PFAAs, Europe and the United States have set concentration limits or health guidelines for certain PFAA concentrations in drinking water. In recent years, the health guidance values for PFAAs have continued to decrease. For example, the Environmental Protection Agency (EPA) in the United States proposed drinking water health advisories for the mix of PFOA and PFOS of 70 ng/L in 2016, which is several orders of magnitude higher than the recently proposed concentration limits of 0.004 ng/L (PFOA) and 0.02 ng/L (PFOS) in 2022. As a result of the annual decrease in lifetime guidance values, the concentrations of PFAAs in surface water and groundwater are gradually approaching or even falling below these standards. For example, in 2022, Cheng et al. detected 31 types of PFAAs in the Qiantang River and the waters of Hangzhou Bay, with PFOA concentrations ranging from 8.8 to 130 ng/L and PFOS concentrations ranging from 1.7 to 24 ng/L, both significantly higher than the EPA standards [11]. Thus, although certain PFAAs are subject to strict regulations regarding production and usage [12,13], complete elimination or replacement within a short timeframe is not feasible [14,15].

The effects of PFAAs in humans encompass a range of health impacts, including impaired fetal growth, histopathological changes, reproductive toxicity, disrupted neurodevelopment and metabolomic responses, cardiometabolic disorders, and abnormal immune system and inflammatory activities [16]. The exposure to PFAAs can induce immunotoxicity in immune organs such as the spleen, bone marrow, and thymus, as well as affect both non-specific and specific immune responses [17]. The presence of PFAAs is also strongly associated with the incidence of immune disorders. For example, Timmermann et al. [18] have identified correlations between elevated serum concentrations of PFOS and PFOA and reduced levels of measles antibodies. Similarly, Kielsen et al. [19] observed negative correlations between PFAA levels and the rate of antibody responses in adults who received tetanus and diphtheria-enhanced vaccine. There is also a close relationship between PFAAs and rheumatoid arthritis [20,21,22].

Sjogren’s syndrome (SS) is a chronic autoimmune disease primarily affecting the exocrine glands, characterized by lymphocytic infiltration and the destruction of glandular tissue [23]. Studies have shed light on the global prevalence of SS, which ranged from 0.04% to 4.8% [24], with China reporting a rate of approximately 0.45% [25]. As research advances, it has been discovered that although individuals with a familial history of the disease may possess an increased inherent susceptibility to the disease, exposure to various environmental factors such as viral infections or chemicals can impact the immune dysfunction and inflammatory response characteristic of SS [26]. C-reactive protein (CRP), erythrocyte sedimentation rate (ESR), immunoglobulins G, A, M (IgG, IgA, IgM), antinuclear antibodies (ANA), anti-Ro52 antibodies (Anti-Ro52), anti-SSA antibodies (Anti-SSA), and anti-SSB antibodies (Anti-SSB) constitute the primary laboratory parameters utilized for the clinical diagnosis of SS [27]. Elevated levels of CRP, ESR, IgG, IgA, and IgM generally indicate the presence of infection and inflammation in the patient’s body and may be used to assess the severity of SS.

Sjogren’s syndrome is also a typical autoimmune disease, which may be closely related to PFAAs. In this study, we recruited a case–control cohort from Hangzhou, China. The objectives of this study were to investigate (1) the statistical characteristics of PFAAs in the serum of the participants, (2) the association between PFAAs and the risk of SS, and (3) the association between PFAAs and SS-related immune parameters.

## 2. Materials and Methods

### 2.1. Study Population and Data Collection

In this study, we initially enrolled 162 patients diagnosed with SS from the Second Affiliated Hospital of Zhejiang University Medical College in Hangzhou, Zhejiang Province, China. The diagnosis of SS in all case groups was based on the classification criteria jointly introduced by the American College of Rheumatology and the European League Against Rheumatism in 2016. Briefly, subjects who presented with dry mouth and dry eyes for more than three months, while excluding active hepatitis C virus infection and acquired immune deficiency syndrome (AIDS), were scored according to different weights. A diagnosis of SS can be made if the total score is greater than or equal to four [28]. The collection of patients was limited to females due to the significantly higher prevalence of Sjogren’s syndrome in this gender, with female patients outnumbering male patients by more than 23 times [29]. Additionally, 156 volunteers were randomly recruited as a control group for analysis. The specific procedures for the inclusion and exclusion of the case and control groups are illustrated in Figure S1. Firstly, the control group participants must meet the following minimum criteria: (1) long-term residency in Hangzhou, (2) no history of SS or other rheumatic diseases, and (3) absence of family history of SS. Secondly, Sjogren’s syndrome can be classified into primary (pSS) and secondary (sSS) [30]. All subjects in the case group were diagnosed with pSS in this study, as patients with sSS had other confirmed rheumatic conditions that could have influenced the final results. Additionally, 34 participants were excluded due to incomplete demographic information (*n* = 18) or lack of serum sample/PFAA data (*n* = 16). All participants were provided with detailed information regarding the specific content and purpose of this study and voluntarily signed a written informed consent form prior to participation. Subsequently, paramedics collected their basic information and serum samples. Basic information on the study population included nine indicators: age, body mass index (BMI), smoking and drinking habits, education level, occupation, income, parity and dietary habits. In addition to measuring concentrations of PFAAs, we also measured levels of CRP, ESR, IgG, IgA, IgM, ANA, Anti-Ro52, Anti-SSA, and Anti-SSB. The current study was reviewed and approved by the Ethics Committee of the Second Affiliated Hospital of Zhejiang University School of Medicine.

### 2.2. Determination of Target PFAAs in Serum

Based on the types of PFAAs commonly detected in Hangzhou City’s population previously [20], this study initially selected perfluoroalkyl carboxylates (PFCAs) with 7 to 14 carbon atoms (perfluoroheptanoate (PFHpA), PFOA, perfluorononanoate (PFNA), perfluorodecanoate (PFDA), perfluoroundecanoat (PFUdA), perfluorododecanoate (PFDoA), PFTrDA, and Perfluorotetradecanoic Acid (PFTeDA)) and perfluoroalkyl sulfonates (PFSAs) with 4, 6, and 8 carbon atoms (perfluorobutane sulfonate (PFBS), perfluorohexane sulfonate (PFHxS), and PFOS). All standards and their internal standard substances used in this experiment were purchased from Wellington Laboratories, Canada.

The ion pair liquid–liquid extraction method was slightly modified and applied for the determination of PFAAs in serum [31]. Shortly, 0.5 ng of internal standards, 2 mL of Na_2_CO_3_/NaHCO_3_ buffer solution (0.25 mol/L), 1 mL of Tetrabutylammonium Hydrogen Sulfate (0.5 mol/L), and 4 mL of methyl tert-butyl ether (MTBE) were added sequentially to 200 μL of serum. The mixture was vigorously shaken for 30 min, followed by centrifugation at a speed of 4000 rpm for 20 min. The resulting supernatant was carefully transferred to another centrifuge tube. Next, add 4 mL of MTBE to the initial tube and perform a second extraction. Finally, evaporate the resulting supernatant from both extractions under nitrogen until dryness and redissolve with 200 μL of methanol.

The target PFAAs were quantified using ultra-performance liquid chromatography ACQUITY UPLC I-Class system coupled with Xevo TQ-S triple quadrupole mass spectrometry. Chromatographic peaks were separated by an Acquity BEH C18 column (75 mm × 2.1 mm, 1.7 μm, Waters, agilent, Santa Clara, CA, USA), while liquid chromatography–mass spectrometry (LC-MS) grade Merck methanol (phase A1) and 2 mM ammonium acetate solution (phase B1) served as mobile phases for phase separation. The detailed conditions of liquid and mass spectrometry are provided in Table S1.

To ensure the stability and reliability of assay data, a process blank and a standard quality control (QC) sample were added to each batch of samples. The accuracy and precision of the assay were monitored by analyzing the concentrations of QC samples. PFAAs with detection rates below 75% were excluded using a triple signal-to-noise ratio as the limit of detection (LOD, 0.05 ng/mL). Concentrations below LOD were replaced by LOD/2.

### 2.3. Statistical Analysis

Since the male-to-female ratio of Sjogren’s syndrome patients has been reported to be quite different [32], considering that gender or sex hormones may have great changes in the evaluation of PFAAs, gender and other related factors were finally excluded from the model.

The data were analyzed using IBM SPSS 26.0 (Softonic, Barcelona, Catalonia, Spain). For continuous variables, we computed and presented their ranges, means, and interquartile concentrations. Mann–Whitney U tests were used to compare continuous variables such as age and BMI, while Chi-square tests were used to compare discontinuous variables such as smoking and drinking habits, education level, occupation, income, parity, and dietary habits. Since the concentrations of PFAAs did not conform to a normal distribution, they were logarithmically transformed using a base-10 scale for subsequent analysis. The results of the normality test for concentrations of PFAAs are presented in Table S2.

Single-factor analysis was used to analyze the significant difference in PFAAs between the case group and the control group, and PFAAs with significant difference was included in the subsequent analysis. We defined significant correlation as *p* < 0.05 and strongly significant correlation as *p* ≤ 0.01. Logistic regression analysis was employed to examine the association between the concentrations of PFAAs and SS, with the discontinuous risk of SS as the outcome variable under evaluation. The adjusted models accounted for age, BMI, smoking and drinking habits, education level, occupation, income, parity, and dietary habits. The evaluation of SS was conducted using *p*-value and 95% confidence intervals. Finally, multivariate linear regression models were employed to examine the association between PFAAs and immunological markers associated with SS in order to investigate the potential impact of PFAAs on SS from an immunological perspective.

## 3. Results

### 3.1. Basic Demographic Information

A total of 136 SS patients and 148 controls were finally included in the subsequent analysis and discussion. The demographic characteristics of the participants are presented in Table 1. Age, BMI, and smoking status data were significantly different between the case and control groups. The mean age of the case group was 52.3 ± 13.0 years, while that of the control group was 48.3 ± 10.8 years old. The patients exhibited a significantly lower body mass index compared to the control group, and their smoking rate was higher in comparison (33.8% vs. 17.6%). There was no significant difference in other demographic data.

### 3.2. Concentrations of PFAAs in Serum

**Figure 1:**
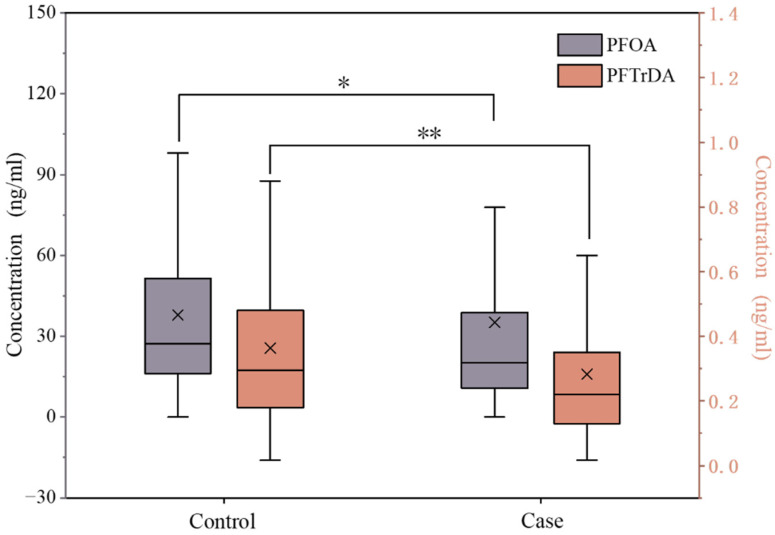
The concentrations of PFAAs in the serum of SS and control. Note: The concentration coordinates for PFOA are on the left and PFTrDA is on the right. X: mean value of each group. * *p* < 0.05, ** *p* ≤ 0.01.

PFAAs with a detection rate above 75% were selected for subsequent correlation analysis, including PFOA, PFNA, PFDA, PFUDA, PFDoA, PFTrDA, PFHxS, and PFOS. As shown in Table S3 and Figure 1, among all the measured PFAAs, the mean concentrations of PFOA in both case and control groups were the highest at 35.19 ± 38.62 ng/mL and 37.85 ± 30.02 ng/mL, respectively, followed by significantly lower levels of PFOS at 14.36 ± 15.73 ng/mL and 15.26 ± 13.64 ng/mL. The average concentration of PFOA was 1.63 ± 1.34 ng/mL in the case group and 1.99 ± 1.68 ng/mL in the control group. The results of single-factor analysis showed that serum concentrations of PFOA and PFTrDA were significantly lower in the case group compared to the control group. No statistically significant differences were observed for the remaining variables under investigation.

### 3.3. Relationship Between Sjogren’s Syndrome and PFAAs

**Figure 2:**
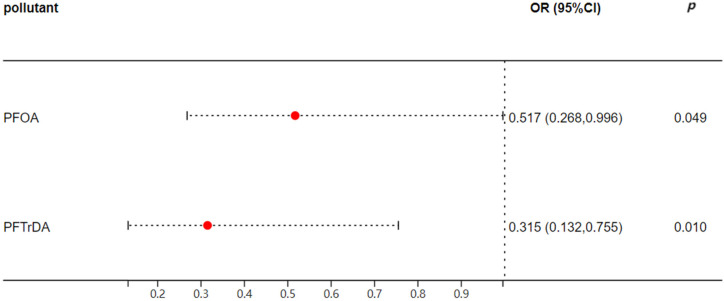
Analysis of correlations between serum PFAAs and SS. Note: Adjusted model was adjusted by age, gender, body mass index (BMI), smoking and drinking habits, education level, occupation, income, parity, and dietary habits. OR, odds ratio. When the OR > 1, it means that the factor is a risk factor; when the OR value < 1, it means that the factor is a protective factor.

After single-factor analysis screening, PFOA and PFTrDA were included in binary logistic regression analysis. As illustrated in Figure 2, the adjusted model reveals that PFOA and PFTrDA exhibit a significant negative correlation with SS. Specifically, each unit increase in log (PFUDA) and log (PFTrDA) may reduce the risk of SS by 48.3% and 68.5%, respectively.

### 3.4. Relationship Between PFAAs and Immune Parameter

**Figure 3:**
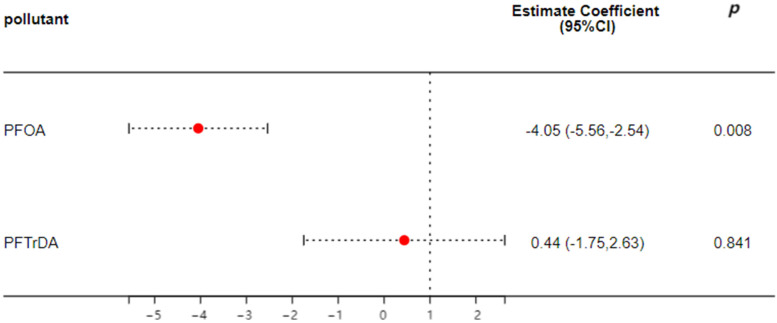
Multiple regression analysis of concentrations of PFAAs and immune and inflammatory markers in the case group. Note: Adjusted model was adjusted by age, body mass index (BMI), smoking and drinking habits, education level, occupation, income, parity, and dietary habits.

Multiple linear regressions were performed to examine the associations between concentrations of PFAAs and immune markers in the case population. The results are presented in Table S4, while Figure 3 illustrates some of the significant correlations. In the adjusted model, PFOA is strongly negatively correlated with CRP, with log-transformed unit increases of −4.05 (95% CI: −5.56, −2.54, *p* = 0.008).

## 4. Discussion

PFOA and PFTrDA were found to be the two PFAAs most closely associated with SS in this study, with detection rates exceeding 75%. This finding implies that these compounds are widely distributed throughout the Hangzhou area, thereby rendering local residents susceptible to their potential impacts. Compared to previous studies on PFAAs in Hangzhou, Jianli Qu et al. [20] observed that the mean concentrations of PFOA and PFTrDA in patients with rheumatoid arthritis were 11.8 and 0.16 ng/mL, respectively, whereas those in the control group were 6.1 and 0.1 ng/mL, respectively. Both concentrations of PFOA and PFTrDA demonstrated an elevation (M_PFOA_ = 35.19 ng/mL, M_PFTrDA_ = 0.28 ng/mL in the case group; M_PFOA_ = 37.85 ng/mL, M_PFTrDA_ = 0.36 ng/mL in the control group), indicating an elevated exposure to PFAAs among Hangzhou residents and a further accumulation of these chemicals within the human population. Compared to PFOA and PFTrDA levels in Zhoukou City, China (M_PFOA_ = 1.59 ng/mL, M_PFTrDA_ = 0.02 ng/mL) [33], Hangzhou exhibited higher concentrations of these compounds due to its status as an eastern coastal city with more developed industries. Consequently, the production and emission of PFAAs are significantly greater than inland areas with fewer developed industry. Compared to the Daegu, Korea (GM_PFOA_ = 3.50 ng/mL, GM_PFTrDA_ = 0.33 ng/mL) [34], Sendai, Japan (GM_PFOA_ = 2.44 ng/mL, GM_PFTrDA_ = 0.60 ng/mL), and Osaka, Japan (GM_PFOA_ = 13.46 ng/mL, GM_PFTrDA_ = 0.52 ng/mL) [35], which are also regions of East Asian countries, higher concentrations of PFOA and lower concentrations of PFTrDA were observed in the serum of Hangzhou residents (GM_PFOA_ = 24.53 ng/mL, GM_PFTrDA_ = 0.26 ng/mL). This outcome can be ascribed to the substantial population base in Hangzhou and the agglomeration of industries associated with PFAAs, as well as the different chain lengths and types used in PFAAs production. Additionally, the serum concentrations of PFOA and PFTrDA in the Shanghai population were marginally lower than those observed in Hangzhou (GM_PFOA_ = 19.6 ng/mL, GM_PFTrDA_ = 0.1 ng/mL) [36], which is a densely populated area adjacent to Hangzhou. Therefore, based on the aforementioned reasons and data, it can be inferred that Hangzhou is confronted with a more severe pollution of PFOA in comparison to numerous other regions. As a result, the population in Hangzhou is also exposed to higher levels of this pollutant.

This study observed a noteworthy association between SS and exposure to PFOA and PFTrDA. It is plausible that long-chain PFAAs have a greater impact on the onset of SS. To date, research investigating the relationship between exposure to PFAAs and autoimmune diseases (AIDs) remains limited. Contrary to the findings of this present study, previous research has predominantly demonstrated a positive correlation between PFAAs, particularly PFOA and AIDs. For example, a study conducted among workers revealed a significant positive correlation between PFOA and two autoimmune diseases, ulcerative colitis and rheumatoid arthritis [37]. Similarly, Jianli Qu et al. [20] found that there was a significant association between rheumatoid arthritis and PFOA (1.998, 95% CI: 1.623, 2.361). Furthermore, a study conducted on mothers residing in areas contaminated with PFAAs revealed that those who were at higher risk of exposure to the chemical reported more health issues and autoimmune diseases (AID), which indirectly suggests the role of certain PFAAs in promoting the risk of AID [38]. According to the aforementioned studies, PFAAs have the potential to disrupt normal immune system functioning and may also contribute to AID.

Previous experimental studies have demonstrated that certain PFAAs can activate nuclear factor-κB and limit the expression of leukocyte chemotactic and adhesion molecules, thereby reducing cytokine release and inhibiting inflammation [39]. In our data, the inverse correlations observed between PFOA and CRP lend support to a potential anti-inflammatory effect of PFOA. Our findings are consistent with several previous animal studies and in vitro cellular studies that have reported negative associations between PFOA and immune or inflammatory factors such as cytokines, immunoglobulins, and immune cells [40,41]. In articles assessing immune function markers in adults and the elderly, a C8 health study based on six PFAA-contaminated waters in Ohio and West Virginia consistently reported significant negative correlations between PFOA and TNF-α, thus confirming the potential anti-inflammatory effects of PFOA [42]. Similar negative correlation results were obtained in a cross-sectional study that also encompassed middle-aged and elderly populations [39]. Therefore, the presence of PFOA may potentially impact the initiation or intensity of SS by modulating the inflammatory response.

However, the impact of PFAAs on inflammatory conditions may not fully explain their effect on SS alone. Therefore, we conducted an analysis of the influence of PFAAs on immune cells and related factors. In an experiment assessing the toxicity exposure of PFOA in mice, a certain degree of impact was observed on the composition or quantity of B cells in both male and female subjects. The author concludes that the development and survival of B cells may be impeded by exposure to PFOA [43]. The involvement of B-cell in SS pathogenesis has been increasingly demonstrated [44], suggesting that exposure to PFOA may play a role in the development of SS by inhibiting the number of B cells.

However, it is worth noting that the serum samples were collected after the onset of SS, and there was a delay in the detection of PFAA concentration. Therefore, the possibility of the reverse causality hypothesis cannot be ruled out, as the disease itself may have had a greater impact on serum PFAA concentrations. Future research on the effects of environmental pollutants and diseases should be conducted in a more comprehensive manner, encompassing disease prevalence during the healthy phase, human immune resistance processes in the initial phase of disease, and disease severity post-disease. Later studies could also focus on the effects of long-chain PFAAs on SS.

## 5. Conclusions

This study analyzed serum samples from 136 SS patients and 148 healthy individuals, finding the highest concentration of PFOA followed by PFOS. Certain PFAAs in serum were significantly associated with the risk of SS, including negative correlations between PFOA/PFTrDA and the risk of SS. Ultimately, we also observed that specific PFAAs exert an impact on inflammatory and immune markers associated with SS. This case–control trial suggests that exposure to PFAAs may influence the risk of SS, but further experiments and studies are required to confirm these causal findings.

## Figures and Tables

**Table 1:** Demographic information of participants.

Characteristics	Cases (*n* = 136)	Controls (*n* = 148)	*p*-Value ^a^
Age (years, mean ± standard)	52.3 ± 13.0	48.3 ± 10.8	0.003
BMI (kg/m^2^)			<0.001
<18.5	23 (16.9%)	24 (16.2%)	
18.5~25	101 (74.3%)	67 (45.3%)	
25~30	10 (7.4%)	37 (25.0%)	
>30	2 (1.5%)	20 (13.5%)	
Smoke			0.002
Yes	46 (33.8%)	26 (17.6%)	
No	90 (66.2%)	122 (82.4%)	
Drink			0.124
Yes	33 (24.3%)	25(16.9%)	
No	103 (75.7%)	123(83.1%)	
Education			0.504
High school and below	103 (75.7%)	117 (79.1%)	
Bachelor or college degree	33 (24.3%)	31 (20.9%)	
Occupations			0.582
Farmers	70 (51.5%)	81 (54.7%)	
Staff and workers	66 (48.5%)	67 (45.3%)	
Income (CNY)			0.101
≤100,000	95 (69.9%)	116 (78.4%)	
>100,000	41 (30.1%)	32 (21.6%)	
Parity			0.203
≤1	70 (51.5%)	65 (43.9%)	
>1	66 (48.5%)	83 (56.1%)	
Eating habits			0.217
Normal	98 (72.1%)	116 (78.4%)	
Love spicy food	38 (27.9%)	32 (21.6%)	

Note: ^a^ The concentrations of age and BMI were compared using Mann−Whitney U tests, while smoking and drinking habits, education level, occupation, income, parity, and dietary habits were compared using Chi-square tests.

## Data Availability

The original contributions presented in the study are included in the article and Supplementary Materials, further inquiries can be directed to the corresponding author.

## Supplementary Materials

The following supporting information can be downloaded at: https://www.mdpi.com/article/10.3390/toxics12100764/s1, Table S1. Detailed liquid and mass spectrometry conditions; Table S2. The results of the normal distribution test for the concentrations of perfluoroalkyl acids; Table S3. The concentration of PFAAs in serum; Table S4. Multiple regression analysis of concentrations of perfluoroalkyl acids and immune and inflammatory markers in the case group; Figure S1. The specific inclusion and exclusion criteria for the case and control groups.

## Abbreviations

ANA	Antinuclear antibodies
Anti-Ro52	Anti-Ro52 antibodies
Anti-SSA	Anti-SSA antibodies
Anti-SSB	And anti-SSB antibodies
BMI	Body mass index
CRP	C-reactive protein
EPA	Environmental Protection Agency
ESR	Erythrocyte sedimentation rate
LC-MS	Liquid chromatography–mass spectrometry
LOD	Limit of detection
MTBE	Methyl tert-butyl ether
PFAAs	Perfluoroalkyl acids
PFBS	Perfluorobutane sulfonate
PFCAs	Perfluoroalkyl carboxylates
PFDA	Perfluorodecanoate
PFDoA	Perfluorododecanoate
PFHpA	perfluoroheptanoate
PFHxS	Perfluorohexane sulfonate
PFNA	Perfluorononanoate
PFOA	Perfluorooctanoate
PFOS	Perfluorooctanesulfonate
PFSAs	Perfluoroalkyl sulfonates
PFTeDA	Perfluorotetradecanoic acid
PFTrDA	Perfluorotrdecanoate
PFUdA	Perfluoroundecanoat
pSS	Primary Sjogren’s syndrome
QC	Quality control
SS	Sjogren’s syndrome
sSS	Secondary Sjogren’s syndrome
