# Supplementary material for: Relationship Between Perfluoroalkyl Acids in Human Serum and Sjogren’s Syndrome: A Case–Control Study of Populations in Hangzhou, China
Source: Toxics. 2024 Oct 21;12(10):764. doi: 10.3390/toxics12100764 (PMC11511288; doi:10.3390/toxics12100764)
Supplement: Supplementary file 1 [file toxics-12-00764-s001.zip › toxics-3203408-supplementary.pdf]

Relationship Between Perfluoroalkyl Acids in Human Serum and Sjogren's Syndrome: A Case–Control Study of Populations in Hangzhou, China

Yun Zhao 1,†, Shetuan Hu 2,†, Hangbiao Jin 2, Chuanbing Fan 2, Kaizhen Liao 2, Songzhao Zhang 3 and Jing Xue 1,*

1 Department of Rheumatology, Zhejiang University School of Medicine Second Affiliated Hospital,
Hangzhou 310009, China; 2315115@zju.edu.cn
2 Key Laboratory of Microbial Technology for Industrial Pollution Control of Zhejiang Province, College of En-vironment, Zhejiang University of Technology, Hangzhou 310032, China; shetuanhu001@163.com (S.H.); hangbiao102@163.com (H.J.); chuanbingfan001@163.com (C.F.); kaizhenliao001@163.com (K.L.)
3 Department of Laboratory Medicine, Zhejiang University School of Medicine Second Affiliated Hospital, Hangzhou 310009, China; 2196005@zju.edu.cn
* Correspondence: jingxue@zju.edu.cn
† These authors contributed equally to this work.

Table of contents

Table S1. Detailed liquid and mass spectrometry conditions

Table S2. The results of the normal distribution test for the concentrations of perfluoroalkyl acids

Table S3. The concentration of PFAAs in serum

Table S4. Multiple regression analysis of concentrations of perfluoroalkyl acids and immune and inflammatory markers in the case group

Figure S1. The specific inclusion and exclusion criteria for the case and control groups

Table S1. Detailed liquid and mass spectrometry conditions

**Mobile phase**	**Milli-Q water (containing 2 mM ammonium acetate) (A) and methanol (B)**			
**Gradient**	Time (min)	Flow rate (mL/min)	A (%)	B (%)
	0.0	0.3	5	95
	1.0		25	75
	5.0		75	25
	9.0		90	10
	10.0		90	10
	10.1		5	95
	12.0		5	95
**Injection volume**	2 μL			
**Analyzed substances**	Parent ion	Daughter ion	Cone voltage (V)	Collision energy (V)
**PFHpA**	363	319	30	10
**PFOA**	413	369	30	10
**PFNA**	463	419	30	10
**PFDA**	513	469	12	10
**PFUDA**	563	519	30	10
**PFDoA**	613	569	18	10
**PFTrDA**	663	619	20	12
**PFTeDA**	713	669	20	12
**PFBS**	299	80	40	30
**PFHxS**	399	80	45	33
**PFOS**	499	99	30	38

Table S2. The results of the normal distribution test for the concentrations of perfluoroalkyl acids

Target compounds	K-S test p-value[a]			S-W test p-value[b]		
	**Total**	**Case**	**Control**	**Total**	**Case**	**Control**
**PFOA**	<0.001	<0.001	<0.001	<0.001	<0.001	<0.001
**PFNA**	<0.001	<0.001	<0.001	<0.001	<0.001	<0.001
**PFDA**	<0.001	<0.001	<0.001	<0.001	<0.001	<0.001
**PFUDA**	<0.001	<0.001	<0.001	<0.001	<0.001	<0.001
**PFDoA**	<0.001	<0.001	<0.001	<0.001	<0.001	<0.001
**PFTrDA**	<0.001	<0.001	<0.001	<0.001	<0.001	<0.001
**PFHxS**	<0.001	<0.001	<0.001	<0.001	<0.001	<0.001
**PFOS**	<0.001	<0.001	<0.001	<0.001	<0.001	<0.001

Note: The normal distribution tests for the concentrations of perfluoroalkyl acids in serum were performed separately using Kolmogorov-Smirnov test and Shapiro—Wilk test.

Table S3. The concentration of PFAAs in serum

Target PFAAs	Case					Control				
	mean ±SD	25	50	75	Range	mean ±SD	25	50	75	Range
**PFOA**	35.19 ±38.62	10.75	20.12	38.96	0.07~ 189.62	37.85 ±30.02	16.10	27.30	51.89	<LOD~ 128.09
**PFNA**	2.75 ±2.60	1.35	1.92	3.37	0.33~ 23.90	3.14 ±2.58	1.44	2.67	4.05	<LOD~ 20.80
**PFDA**	2.44 ±2.49	1.00	1.68	2.98	0.16~ 18.15	2.66 ±2.55	1.20	1.88	3.12	0.16~ 15.40
**PFUDA**	1.63 ±1.34	0.78	1.28	2.02	<LOD~ 9.24	1.99 ±1.68	0.88	1.47	2.37	0.07~ 9.69
**PFDoA**	0.23 ±0.22	0.10	0.17	0.26	<LOD~ 1.44	0.21 ±0.18	0.10	0.15	0.28	<LOD~ 0.91
**PFTrDA**	0.28 ±0.22	0.13	0.22	0.35	<LOD~ 1.47	0.36 ±0.26	0.18	0.30	0.49	<LOD~ 1.66
**PFHxS**	3.14 ±3.37	0.96	1.90	3.91	0.10~ 19.18	3.50 ±5.15	1.26	2.51	4.30	0.11~ 57.23
**PFOS**	14.36 ±15.73	3.80	8.80	20.02	<LOD~ 75.04	15.26 ±13.64	5.92	11.63	19.26	<LOD~ 66.28

Note: Concentration unit: ng/mL. <LOD: The concentration is lower than the detection limit, 0.05 ng/mL.

Table S4. Multiple regression analysis of concentrations of perfluoroalkyl acids and immune and inflammatory markers in the case group

	**PFOA (95% CI)**	**PFTrDA (95% CI)**
**CRP**	-4.05 (-5.56,-2.54)*	0.44(-1.75,2.63)
**ESR**	-7.83(-12.36,-3.30)	-2.79(-9.34,3.76)
**IgG**	-0.43(-1.24,0.38)	-0.85(-2.02,0.32)
**IgA**	8.76(3.89,13.63)	0.98(-6.06,8.02)
**IgM**	0.00(-0.13,0.13)	0.26(0.07,0.45)
**ANA**	13.77(-0.93,28.47)	19.1(-2.16,40.36)
Anti-**Ro52**	-2.89(-6.8,1.02)	-4.01(-9.66,1.64)
Anti-**SSA**	5.51(-0.01,11.03)	-8.33(-16.32,-0.34)
Anti-**SSB**	6.23(2.84,9.62)	-2.73(-7.63,2.17)

Note: CI, confidence interval. Adjusted by age, BMI, smoking and drinking habits, education level, occupation, income, parity and dietary habits. All concentrations of perfluoroalkyl acids (ng/mL) and immune and inflammatory markers concentrations were log-transformed. *p-value < 0.05, **p-value ≤ 0.01.

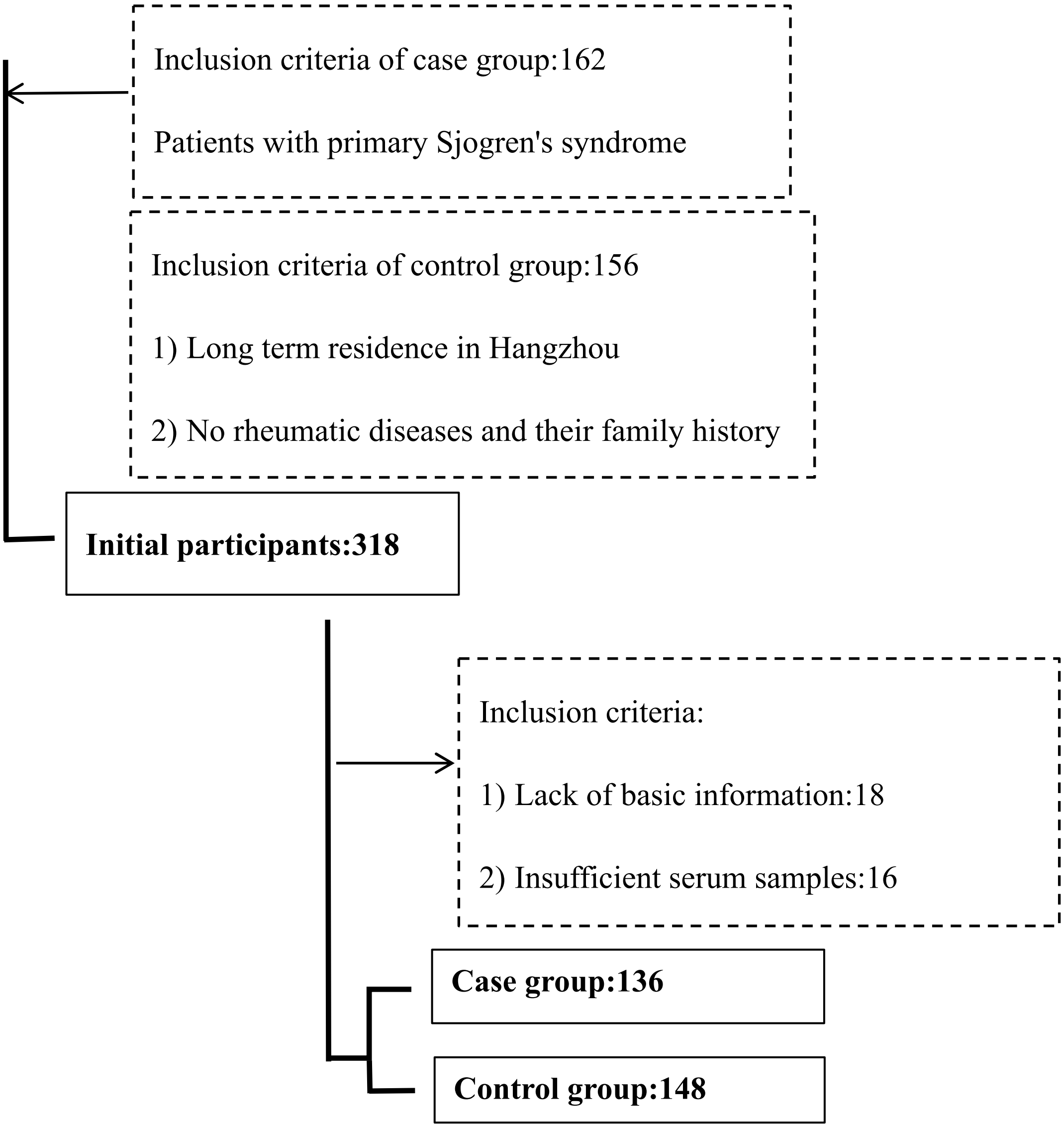

Figure S1. The specific inclusion and exclusion criteria for the case and control groups
